# Multiplex detection and identification of viral, bacterial, and protozoan pathogens in human blood and plasma using an expanded high-density resequencing microarray platform

**DOI:** 10.3389/fmolb.2024.1419213

**Published:** 2024-06-20

**Authors:** Moussa Kourout, Scott Espich, Carolyn Fisher, Irina Tiper, Anjan Purkayastha, Sean Smith, Luis Santana-Quintero, Robert Duncan

**Affiliations:** ^1^ Division of Emerging and Transfusion Transmitted Diseases, Office of Blood Research and Review, Center for Biologics Evaluation and Research, Food and Drug Administration, Silver Spring, MD, United States; ^2^ Openbox Bio, Vienna, VA, United States; ^3^ HIVE Team, Office of Biostatistics and Pharmacovigilance, Center for Biologics Evaluation and Research, Food and Drug Administration, Silver Spring, MD, United States

**Keywords:** microarray, blood-borne pathogens, multiplex detection, targeted sequencing, resequencing microarray

## Abstract

**Introduction:** Nucleic acid tests for blood donor screening have improved the safety of the blood supply; however, increasing numbers of emerging pathogen tests are burdensome. Multiplex testing platforms are a potential solution.

**Methods:** The Blood Borne Pathogen Resequencing Microarray Expanded (BBP-RMAv.2) can perform multiplex detection and identification of 80 viruses, bacteria and parasites. This study evaluated pathogen detection in human blood or plasma. Samples spiked with selected pathogens, each with one of 6 viruses, 2 bacteria and 5 protozoans were tested on this platform. The nucleic acids were extracted, amplified using multiplexed sets of primers, and hybridized to a microarray. The reported sequences were aligned to a database to identify the pathogen. To directly compare the microarray to an emerging molecular approach, the amplified nucleic acids were also submitted to nanopore next generation sequencing (NGS).

**Results:** The BBP-RMAv.2 detected viral pathogens at a concentration as low as 100 copies/ml and a range of concentrations from 1,000 to 100,000 copies/ml for all the spiked pathogens. Coded specimens were identified correctly demonstrating the effectiveness of the platform. The nanopore sequencing correctly identified most samples and the results of the two platforms were compared.

**Discussion:** These results indicated that the BBP-RMAv.2 could be employed for multiplex detection with potential for use in blood safety or disease diagnosis. The NGS was nearly as effective at identifying pathogens in blood and performed better than BBP-RMAv.2 at identifying pathogen-negative samples.

## Introduction

Safety of blood and blood products is the priority for transfusion and tissue transplantation. The risk of transfusion-transmitted viral infections is significantly reduced by the implementation of nucleic acid tests (NATs) in parallel with conventional methods such as serological tests and questionnaires for blood donor screening (Coste et al., 2005; Roth et al., 2012). Currently, NATs are the routine approach for testing blood components and tissue transplants for HIV, HCV, HBV, and West Nile Virus (WNV) in the US using the mini-pool strategy (Chamberland et al., 2001; Coste et al., 2005). Emerging viral infections have caused blood safety responses to Zika virus. Recently, NAT testing has been applied to protozoan parasites (Tonnetti et al., 2022).

Although these tests have successfully reduced the risk and diagnostic window periods, single-donor testing could further improve sensitivity, which is important for pathogens at low concentration. The goal is to enable the detection, all in one single test, of additional organisms that threaten the blood supply, of which there are many (Stramer et al., 2009). Viruses like chikungunya and dengue as well as new strains of bacteria and parasites (*Plasmodium falciparum* and *Babesia microti*), other variants of already known viruses (HIV, HCV, and HBV) that could potentially escape detection with the existing blood donor screening tests, and many other non-routine blood-transmissible agents are all causes of concern. Therefore, the development of alternative blood screening tests detecting multiple pathogens from one single specimen, without losing the sensitivity and accuracy of conventional techniques, is now a priority for improving blood safety.

Research studies propose that microarrays can resolve a large number of targets usually amplified from the specimen by PCR, (Tomioka et al., 2005; Hsia et al., 2007; Lung et al., 2017; De Giorgi et al., 2019), showing promise in pathogen detection. Other platforms have used spatially multiplexed real-time PCR (Grigorenko et al., 2014).

Unlike the printed oligonucleotide arrays used mostly for gene expression experiments (Palmer et al., 2006; Wong et al., 2007), the Affymetrix GeneChips have been adapted for sequencing by hybridization of target DNA fragments. We previously published a study utilizing the *in situ*-synthesized GeneChips with high-density arrayed oligonucleotide probes to detect and identify blood-borne pathogens (Kourout et al., 2016). The previous study was conducted to assess the feasibility of the resequencing microarray (RMA) platform in testing blood donors.

The current study expands the platform to achieve low concentration detection and the unmatched specificity of determining the actual sequence of the targeted pathogens. There are new probes on the chip for segments of pathogen genomes that target more blood-borne pathogens with higher discrimination of species, strains, and genotypes. The blood-borne pathogen resequencing microarray version 2 (BBP-RMAv.2) was evaluated for its ability to detect and identify viral, bacterial, and protozoan pathogens spiked in blood or plasma that were selected because they are of concern for blood safety and were not tested on the previous version.

The current molecular detection methods are increasingly turning to next-generation sequencing (NGS) for accurate identification of multiple pathogens on one platform (Chiu et al., 2015). To compare the results of NGS to those of the BBP-RMAv.2, PCR products prepared in the microarray workflow were also used for library preparation and sequencing on the Oxford Nanopore MinION (Lin et al., 2021). This allowed evaluating the strengths and weaknesses of each method and side-by-side comparison.

## Materials and methods

### BBP-RMAv.2 design

**FIGURE 1:**
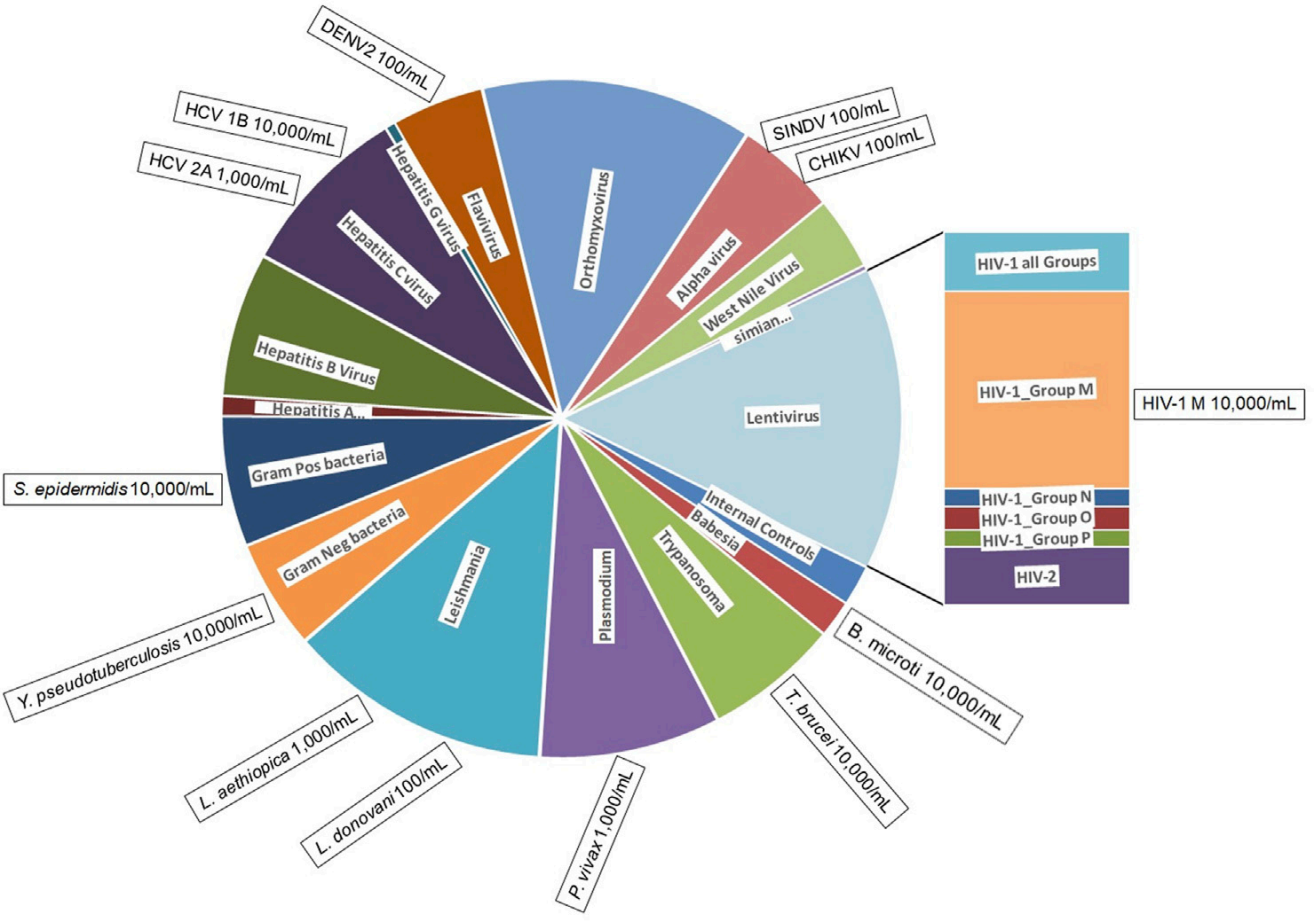
BBP-RMAv.2 microarray composition and pathogens detected. The BBP-RMAv.2 chip design contains 179 tile detectors of target sequence regions. The chip was designed to detect and identify 80 different organisms. The chip allocates two control tile detectors (NAC1 and TIM) and 177 pathogen tile detectors interrogating a total of 47,973 nucleotides of target pathogen genomic sequences; these cover a wide range of pathogens distributed among viruses, bacteria, and protozoans. The pie chart is scaled to the number of tiled bases in each category of pathogen (pie wedge). This microarray assay was evaluated for the detection and identification of 13 pathogens, indicated in the boxes positioned at the perimeter outside the category they belong to along with minimum concentrations detected, illustrating to what extent testing covered the range of pathogen types.

The BBP-RMAv.2 was designed to identify 80 different pathogens categorized in 16 groups (Figure 1). Each pathogen sequence is represented by one or more locations on the microarray. The locations, called tile detectors, are prototype sequences that correspond to the targeted regions in the pathogens (Supplementary Table S1). Each tile detector comprises a series of overlapping oligonucleotide probes that are 25 bases long. Sets of four probes (one perfect-match probe and three probes with one of the other three mismatch bases in the 13th position) were generated for each of the bases in the prototype sequences (Gingeras et al., 1998), which range in length from 45 to 876 bases along the length of the pathogen genome. There are 177 detector tiles that cover a total of 47,973 nucleotides. Sequencing by hybridization requires a total of 383,784 oligonucleotide probes (including both strands) on the microarray to interrogate all the bases. For target regions in pathogens that are highly variable, multiple detector tiles were designed for the same sequence region, with each tile composed of probes corresponding to a particular strain or variant. For example, the 17 HIV-1 LTR tiles cover the same gene sequence, with each one designed to match the sequence of a different variant. The tile detector design was sent to Affymetrix, Inc. (Santa Clara, CA) for manufacturing, as previously described (Lin et al., 2006).

The complete description of the microarray is available at ArrayExpress (https://www.ebi.ac.uk/biostudies/arrayexpress/arrays/A-MTAB-670) with the accession number A-MTAB-670. Note that the archive is labeled as Ebolavirus Resequencing Array, however, the array contains probes for the blood-borne pathogens as well.

### Primer design and optimization of primer pools

Specific primers were designed to enable PCR amplification of genome regions matching the target tiles that are on the array (Supplementary Table S2). Primers were extended with a primer–linker sequence at the 5′ end (Supplementary Table S2), and a linker–primer was added to the amplification mix to minimize dimer formation and enhance PCR amplification of the selected targets (Brownie et al., 1997). All steps of primer design, selection, and pool assembly were carried out as previously described (Kourout et al., 2016). Primers that were amplified in a single PCR were checked for efficient function in the multiplex PCR. Mixtures of primer pairs were assembled into four separate primer pools, with 8–12 pairs in each, using the “Multiplex All Sets” tool in the software OLIGO, version 7.60 (Molecular Biology Insights, Inc., Cascade, CO). Primer pools were tested under multiplex PCR conditions.

### Specimen collection and sample processing

Chikungunya virus, hepatitis C virus genotype 1b, and HIV-1 group M samples were prepared from the CBER Lot Release panel members with copies/mL extensively validated by CBER (Grigorenko et al., 2014). The hepatitis C virus genotype 2a (HCV2a) is a virus stock of the strain J6/JfH1 (Duan et al., 2010). HCV genome copies/mL was established by quantitative PCR (Applied Biosystems 7500 RT-PCR Guide, PN 4347825). Dengue virus serotype 2 (DENV2) and Sindbis virus were laboratory stocks quantified by qPCR as PCR detectable units (PDU) per mL. To calculate PDUs, the nucleic acid was extracted from the original virus stock, and then 10-fold serial dilutions were made in the buffer. Each dilution was used as a template in singleplex real-time PCRs utilizing the same primers that were added to the multiplex pools. The lowest dilution that gave a Ct value at least two cycles less than the no-template control was assigned the value of one PCR detectable unit. The inverse of that dilution was the number of PDUs in the undiluted sample, and from that, the PDUs/mL was calculated.

*Babesia microti* (ATCC PRA-99) parasite cells were collected from an infected DBA/2 mouse at a level of microscopically determined parasitemia of 10%–15%. The *Babesia* cells/mL was calculated by microscopic determination of the percent of infected red blood cells (RBCs) and the determination of the number of RBCs per mL with a particle counter (Beckman/Coulter), and then the *Babesia* cells/mL was calculated by the product of these two numbers. Subsequently, infected mouse red cells were diluted in whole human blood to the desired concentrations. *Leishmania aethiopica* was purchased from ATCC (Manassas, VA), catalog number 50119, lot 125497, at a concentration of 1.1 × 10^6^ cells/mL. *Leishmania donovani* 1S2D promastigote form (Debrabant et al., 2004) and *Trypanosoma brucei* as epimastigotes (Selvapandiyan et al., 2007) were cultured as previously described and quantified by automated microscopic image analysis (Vision 286 Cellometer, Nexcelom Bioscience, Lawrence, MA). *Plasmodium vivax* was obtained from ATCC (Manassas, VA) catalog number 30152, lot 3714526, labeled 5.0 × 10^6^ parasites per 0.5-mL vial.

Bacterial species *Yersinia pseudotuberculosis* and *Staphylococcus epidermidis* were cultured in super broth (Quality Biological Inc., Gaithersburg, MD) and quantified by measuring OD_600_ and converting to cells/mL with the formula 5.0 × 10^8^ cell/mL = 1 absorbance unit in a spectrophotometer (SmartSpec 3000, Bio-Rad Laboratories, Inc., Hercules, CA). This spectrophotometric method was shown to result in a cell count 100 times higher than a plate count for colony-forming units. However, the copy number measured by quantitative PCR is closer to the spectrophotometric measurement, reflecting the enumeration of cells that are not viable (Dong et al., 2016). The spiking range used for the molecular detection method in this study was best standardized to the spectrophotometric method.

All bacterial and protozoan pathogens were similarly diluted and spiked into whole heparinized pathogen-negative human blood. Viral pathogens were diluted and spiked into pathogen-negative human plasma.

### Ethics statement

The studies involving humans were approved by FDA Research Involving Human Subject Committee and the National Institutes of Health Institutional Review Board. The studies were conducted in accordance with the local legislation and institutional requirements. The participants provided their written informed consent to participate in this study. No potentially identifiable images or data are presented in this study.

### BBP-RMAv.2 processing

The BBP-RMAv.2 assay protocol is a series of steps described in our previous publication (Kourout et al., 2016) and briefly summarized in the following paragraphs.

Nucleic acids from plasma spiked with viral pathogens and DNA from whole blood specimens were extracted, and the appropriate volume was added to a reverse transcription (RT) reaction. The products from the RT reaction were divided among the four PCRs, each containing one of the primer pools with internal positive controls and the primer–linker oligonucleotide. After several rounds of specific amplification, the primer–linker continues the amplification of the existing PCR products. The PCR products were combined and purified with a DNA clean-up system (Wizard SV Gel and PCR Clean-Up System, A9282, Promega, Madison, WI).

Microarray hybridization and processing were carried out according to the manufacturer’s protocol (Affymetrix Inc., Thermo Fisher Scientific, Carlsbad, CA). The scanned microarray image allowed sequence data to be generated as base calls using the GeneChip Sequence Analysis Software (GSEQ, Affymetrix, Inc.). The output is a FASTA file compiling all the detector tiles with the called bases for each tile.

The details of the microarray results including the image file (CEL files), base-calling file (CHP), the final called base sequence (txt files), and the pipeline output “blast.report” of each microarray are available at ArrayExpress (https://www.ebi.ac.uk/arrayexpress/) under the accession number E-MTAB-13972 with the title: “Blood borne pathogen detection in blood and plasma by Microarray” at the link: https://www.ebi.ac.uk/biostudies/arrayexpress/studies?query=E-MTAB-13972+.

### Microarray-derived sequence analysis

The FASTA file was the input for a custom bioinformatic pipeline, *bbp_i2o*. The code for the bbp_i2o pipeline and construction of TVRSD is freely available at: https://github.com/FDA/Resequencing-Microarray-Pipeline/blob/main/bbp_i2o_2. As previously described (Tiper et al., 2022), a very simple graphic user interface allows the choice of the pipeline and the choice of the FASTA file. With a click on the “run” button, the *bbp_i2o* executes a series of PERL-based scripts that link together some minor calculations and open-source software: BLAST for database searching (Altschul et al., 1990) and MUSCLE for multiple sequence alignment (Edgar, 2004), all unseen by the operator. The *bbp_i2o* pipeline is loaded on a Dell Precision Tower 7910 workstation with Dual Intel Xeon Processor E5-2 650 v3, 64 GB memory, and two hard drives (1 TB and 0.5 TB). As a first step, the pipeline determines the C3 score for each tile. The C3 score is the total number of nucleotides that occur in runs of three or more consecutive (non-N) base calls, expressed as a percentage of the length of the tile sequence (in bases) (Metzgar et al., 2010). The C3 score is a measure of the quantity of hybridized DNA and the quality of hybridization. Tile sequences with C3 scores greater than or equal to 20.0 are considered to represent true positive hybridization events; tile sequences with lower scores are postulated to be products of cross-hybridization or assay noise.

Tile sequences that qualify are searched for homologous sequences in a local Validated Reference Sequence Database (TVRSD) (Metzgar et al., 2010) that is stored on the Dell Precision Tower 7910 workstation using the BLAST alignment algorithm. BLAST results for each tile with C3 score ≥20 are placed in a report (blast.report), which contains the top three database hits, i.e., their Bit-score, E-value, accession number, and scientific name, along with the C3 score for each tile. This “blast.report” is deposited into a date and time-stamped folder along with a text file with the original FASTA file of all the tiles and a “blast.html” file that shows all the significant alignments of the top hit tile that is displayed in an internet browser window upon opening.

### Nanopore next-generation sequencing

Samples were processed through extraction, reverse transcription, multiplex PCR reactions, pooling, and cleanup in preparation for the microarray, as described in the “BBP-RMAv.2 Processing” section above. These procedures generate enough nucleic acids to hybridize the microarray and prepare a library for MinION sequencing. Approximately 300 ng of DNA from the pooled PCR product sample was used to prepare a library with the Oxford Nanopore Amplicons-by-Ligation kit (SQK-LSK112) (Oxford_Nanopore_Technologies, 2023) or ligation sequencing amplicons-native barcoding kit (SQKNBD112.24) (Oxford_Nanopore_Technologies, 2021) following the manufacturer’s instructions.

The prepared libraries were loaded onto a MinION Mk1b with a SpotON Flow Cell, version R9 (FLO-MIN106D, Oxford Nanopore Technologies, Oxford, United Kingdom). The MinKNOW software, version 22.03.5 (Oxford Nanopore Technologies, Oxford, United Kingdom) collects the reads, performs base-calling, and sorts the reads into “failed reads” and “passed reads.” After 4 h of read collection, followed by several additional hours of base-calling, the passed reads as fastq.gz files were deposited on the hard drive of a Mac Pro computer. Fastq.gz files were uploaded to the FDA High-Performance Integrated Virtual Environment (CBER_HIVE, 2023), which is a specialized platform developed by CBER’s HIVE staff containing a sequence read archive with direct access to the high-performance computer cluster. Reads from a sequencing run were quality control-checked and aligned against a unique reference file with the alignment tool Minimap2 (Li, 2018), an advanced tool optimized for longer reads typical of nanopore sequencing. The number of reads aligning (mapping quality ≥20) with each pathogen sequence in the reference file is reported.

The reference file is composed of a select group of the sequences present in the tiles arrayed on the BBP-RMAv.2 microarray, listed in Supplementary Table S1. The sequences selected for the reference file are marked with an asterisk (*) in Supplementary Table S1. The reference file also has a human 18S rRNA sequence file, accession number NR_146117.1, available from the NCBI nucleotide database, to reduce false alignment of human sequence reads with protozoan 18S rRNA reference sequences. The sequences for the *B. microti* 18S rRNA tile and the *Leishmania* sp. 18S rRNA were excluded from the reference to reduce cross-alignment.

## Results

We evaluated the BBP-RMAv.2 assay using 13 high-priority blood-borne pathogens to evaluate the detection of nearly all the types of pathogens represented on the microarray (Figure 1). The detection of these pathogens at the species, subtype, and genotype level is assured by a total of 177 tile detectors on the microarray.

### Tile design and multiplex PCR optimization

The target prototype sequences were designed and manufactured as an Affymetrix GeneChip (Figure 1). Tile sequences (Supplementary Table S1) were chosen to optimize sensitivity and discrimination between pathogens. A series of primer testing and optimizations were performed first in single-primer pair reactions. Representative optimization results are shown in Table 1. Once single-primer pair detection of purified nucleic acids was achieved, multiplexed primer pools were tested with purified nucleic acids. For example, the Chikungunya NSP4 primer pair yielded a high-quality sequence on the BBP-RMAv.2 first with purified RNA and then with virus particles in plasma at 1,000 copies/mL. Subsequent testing with virus particles in the plasma at two concentrations with the full multiplex assay is shown in the next section. DENV2, HIV-1, HCV genotype 2a, and HCV genotype 1b virus assays were optimized in a similar fashion (Table 1) with varying sensitivity.

**TABLE 1:** Optimization of the RMA-BBPv.2 using partial assay conditions.

Sample	PCR primers	Tile detector name	Ave. C3^*^ score value at the indicated spiked pathogen copies/mL							
			100,000	10,000	1,000	Nucleic acid		Virus stock		
						pg	C3	Copies	C3	BLAST hit
Chikungunya virus	NSP4 primers only	CHIK_NSP4				100	94.3			CHIKV
Chikungunya virus	NSP4 primers only	CHIK_NSP4			95.67					CHIKV
Dengue virus serotype 2	NS5 primers only	DENV2_NS5			94					DENV2
Dengue virus serotype 2	6–2017 primer pools	DENV2_POLYP						400	62	DENV2
HIV-1 M	GAG primers only	HIV1M_P24	78.1							HIV-1
HCV 2a virus stock	2021 primer pools	HEPCV2_5UTR				4,000	97.9			HCV2a
*Y. pseudotuberculosis*	SUCA and HSP60 primers	YEPS_SUCA				8	94			*Y. pestis*
*S. epidermidis*	2022 pools	STEP_RPOB				8	99			*S. epidermidis*
*B. microti*	2021 primer pools	BAMI_18SRRNA				100	97.8			*B. microti*
*L. donovani*	LEISH 18S rRNA primer pair	TRBR_18SRRNA				100	69			*L. don*
*L. donovani*	2021 primer pools	LEDO_ITS1-2				8	90.7			*L. don*
*T. brucei*	2022 pools	TRBR_ISG75				10	89.6			*T. brucei*
*L. donovani*	2021 primer pools	LESH_18SRRNA	57							*L. don*
*P. vivax*	18S rRNA primers only	PLVI_18SRRNA-2		80						*P. vivax*

^*^ C3 score is a confidence index (scale range from 0 to 100) measuring the detected DNA sequence quality.

Optimization of detection of bacteria species was initiated with purified DNA at a low concentration by testing two primer pairs for targets in *Y. pseudotuberculosis*. Of the two, the SUCA primer pair accomplished near-complete base calls, though the BLAST search showed an identical match in *Y. pseudotuberculosis* and *Yersinia pestis*, which is an undesired suggestion of the presence of this bio-threat agent (see Discussion). SUCA primers were added to the pools due to their sensitivity of detection. The updated pools (Supplementary Table S2) achieved near-perfect sequencing of purified DNA from the Gram-positive bacterium, *S. epidermidis*.

Optimization of detection of protozoan parasites that are found in the blood during an infection began with primer pairs that amplified the 18S ribosomal RNA segments. Earlier multiplex primer pools and individual primer pairs successfully detected purified DNA from *B. microti*, *L. donovani*, and *T. brucei* (Table 1). The same pools detected *L. donovani* diluted in whole blood at 100,000 cells/mL, though the result suggested that amplification needed to be improved. Therefore, additional *L. donovani* primers were added in the final primer pools, resulting in better detection in the final assay.

Once the primer pools were adjusted so that these tests were successful, full assay testing with updated multiplex primer pools was used to detect pathogens in blood or plasma.

### Analytical performance of the BBP-RMAv.2 platform

The analytical detection capability of the BBP-RMAv.2 assay was evaluated using pathogens spiked in blood and plasma samples. Chikungunya virus, hepatitis C virus genotype 1b, and HIV-1 group M specimens were CBER Lot Release Panel members comprising human plasma containing the virus. The Lot Release Panels were diluted in human plasma at 10,000, 1,000, and 100 copies/mL. HCV2a specimens were created from a recombinant virus culture supernatant at 1,000 copies/mL (Table 2). The final optimized (2023) primer pools (Supplementary Table S2) effectively amplified all the virus samples diluted in human plasma, though there were differences in sensitivity. The chikungunya primers and detector tile were effective, though at 100 copies/mL, and the base calls on the NSP4 tile did not align with any database files using the automated pipeline default parameters. No other tiles in the microarray output FASTA file showed base calls, so additional, manual analysis was performed: the C3 score was manually calculated (Table 2), and the sequence of the CHIK_NSP4 tile was submitted to an NCBI BLAST search. BLAST parameters set to “somewhat similar sequence,” “allow six matches in the query range,” word size of seven, and match/mismatch score = 4/−5 resulted in hits on only chikungunya files with bit scores of 56 and E values of 0.004, which was sufficient to interpret the result as a positive detection. The least effective virus detection was of HCV 1b at 10,000 copies/mL (Table 2), suggesting that additional optimization of primers and tile is warranted. The Sindbis virus primers and tile were particularly effective, resulting in all the bases on the SINDBIS_NSP4-1 tile called correctly down to 100 copies/mL.

**TABLE 2:** Analytic performance of the RMA-BBPv.2 (2023 primer pools) using spiked human plasma samples.

Sample^†^	Source	Target region	Tile detector name	Ave. C3^‡^ score value at the concentration spiked copy or PDU/mL				Lowest detected copies/mL
				10,000	1,000	100	0	
CHIKV	CBER/FDA	NSP4	TOGA_CHIK_NSP4	ND^§^	93 **#**1^**^	36^††^	-	100
DENV2	CBER/FDA	NS5	FLAV_DENV2_NS5	ND	84	53 **#2**	-	100
HCV 1b	CBER/FDA	5′ end	FLAV_HEPCV_5UTR	49	0	ND	-	10,000
HCV 2a	CBER/FDA	5′ end	FLAV_HEPCV2A_5UTR	ND	91.7	ND	-	1,000
HIV-1 M	CBER/FDA	GAG	RETR_HIV1M_P24	86 **#3**	0	ND	-	10,000
SINDV	CBER/FDA	5′ end	TOGA_SINDBIS_NSP4-1	100	100 **#4**	100	-	100
Neg **#5** Plasma	NIH donor	-	TRYP_LESH_18SRRNA	-	-	-	21^‡‡^	-
Neg **#6** Plasma	NIH donor	-	HEPCV1_5UTR	-	-	-	30.5^‡‡^	-

^‡^ C3 score is a confidence index (scale range from 0 to 100) measuring detected DNA sequence quality.

^§^ Not done.

^**^ Hashtag numbers indicate a DNA sample that was submitted to MinION sequencing in the same numbered dataset in Table 5 below.

^††^ See text—“manual analysis”.

^‡‡^ See evaluation of false positive in Discussion.

The tile detector C3 scores were high enough to identify the six plasma-spiked viruses and determine the lowest detected concentration of each based on the concentrations tested (Table 2). Plasma samples negative for pathogens were tested with the BBP-RMAv.2 system. One tile for *Babesia* 18S rRNA showed a low number of base calls in the result with this sample. The *Babesia* 18S rRNA is not shown in Table 2 because this consistent false-positive outcome was avoided by eliminating the *B. microti* 18S rRNA tile from the analysis. *Babesia* was adequately detected with the BABE_BAMI_CCT7 tile. Low levels of base calls for *Leishmania* 18S rRNA or HCV in two tested pathogen-negative samples were interpreted as false positives. Detailed analysis of the origin of these undesirable base calls is presented in the Discussion section.

Gram-positive bacteria species *S. epidermidis* and Gram-negative *Y. pseudotuberculosis,* cultured in the laboratory and diluted in whole human blood at 100,000 and 10,000 cells/mL were detected by BBP-RMAv.2, though *S. epidermidis* at 10,000 cells/mL was very close to the cutoff of C3 = 24 (Table 3). Five protozoan parasites (*B. microti*, *L. aethiopica, L. donovani*, *P. vivax*, and *T. brucei*) were diluted in whole human blood at 10,000, 1,000, and 100 cells/mL. All were detected at 10,000 cells/mL, and some at 1,000, but only *L. donovani* was detected at the lowest concentration of 100 cells/mL. The lowest concentration of *L. donovani* gave an initial pipeline BLAST report that selected *L. infantum* for identification of the pathogen in the sample. Further examination of the alignments revealed the tile result was identical in the *L. infantum* genome and the *L. donovani* genome. Thus, at this parasite load, the two closely related species are not distinguished by the microarray sequence result.

**TABLE 3:** Analytic performance of the RMA-BBPv.2 using spiked human blood samples.

Sample^§§^	Source	Target region	Tile detector name	Average C3 score^***^ value at the concentration spiked pathogen cells/mL					Lowest detected (cells/mL)
				100,000	10,000	1,000	100	0	
*S. epidermidis*	ATCC 700562	rpoB	STAP_STEP_RPOB	75 **#14** ^**†††**^	24	0	ND	-	10,000
*Y. pseudotuberculosis* ^*‡‡‡*^	CBER/FDA	sucA	ENTB_YEPS_SUCA	ND	43 **#15**	ND	ND	-	10,000
*B. microti*	CBER/FDA	CCT7	BABE_BAMI_CCT7	ND	92 **#7**	0 **#8**	ND	-	10,000
*L. aethiopica*	ATCC	ITS1	TRYP_LEAE_ITS1-1	ND	82 **#9**	86 **#10**	ND	-	1,000
*L. donovani*	CBER/FDA	18SrRNA	TRYP_LESH_18SRRNA TRYP_LEIN_ITS1	ND	96	86	36^§§§^	-	100
*P. vivax*	ATCC	18SrRNA	HAEM_PLVI_18SRRNA-2	ND	88 **#11**	75 **#12**	ND	-	1000
*T. brucei*	CBER/FDA	18SrRNA	TRYP_TRBR_18SRRNA	ND	29 **#13**	0	ND	-	10,000
Neg whole blood	NIH donor		none	-	-	-	-	0**#16**	-

^§§^ Pathogens spiked in blood.

^***^ C3 score is a confidence index (scale range from 0 to 100) measuring detected DNA sequence quality. Zero indicates non-detection.

^†††^ Hashtag numbers indicate the DNA sample that was submitted to MinION sequencing in the same numbered dataset in Table 5 below.

^‡‡‡^ Tomioka et al., 2005.

§§§ See text for details of post-pipeline manual analysis.

Each pathogen was detected and identified by at least one target sequence tiled onto the BBP-RMAv.2 chip (Table 3). The assay results reveal the actual nucleotide sequences of the detected targets accounting for the usual high level of specificity. The specificity of the BBP-RMAv.2 is also indicated by the lack of cross-reactivity among the seven pathogen-containing whole blood samples and one pathogen-negative whole blood sample that showed no tiles with base calls (Table 3).

### Evaluation of the BBP-RMAv.2 platform using coded whole blood and plasma specimens

With further performance evaluation, the BBP-RMAv.2 was challenged with coded blood and plasma specimens. The coded specimen’s nucleic acids were extracted at the CBER/FDA laboratory, then re-aliquoted into identical tubes, coded, and returned to the lab for testing. The sample identities were revealed only after the results had been finalized.

Three coded plasma nucleic acid samples including the pathogen-negative plasma resulted in two true positives and one false positive (Table 4). One coded blood sample was detected as a true positive (Table 4). Correct identification of unknown samples demonstrates the potential for clinical use of a platform such as the BBP-RMAv.2.

**TABLE 4:** Performance of BBP-RMAv.2 using unknown spiked human blood and plasma.

Spiked pathogen	Conc. Copies/mL	BBP-RMA result	C3 score	Interpretation
CHIKV	1,000	CHIKV	94	True positive
*P. vivax*	10,000	*P. vivax*	60	True positive
Sindbis virus	100	Sindbis virus	93	True positive
Negative Plasma	0	HCV	30.5	False positive

### Comparison of the BBP-RMAv.2 platform to targeted next generation sequencing with the Oxford Nanopore MinION device

To evaluate the comparative effectiveness of a microarray platform to targeted next-generation sequencing for detection of pathogens in blood, identical samples were subject to analysis by each system.

PCR products pooled from the multiplex amplifications of 16 of the samples, indicated by the hashtag numbers in Tables 2, 3, were each used to prepare a library that was loaded in a MinION nanopore sequencing device. The passed reads were aligned against a database or reference file and the number of reads aligning with each pathogen reported. Initial attempts searching the NCBI nucleotide database or a custom-assembled reference file containing the genomes of the 30 pathogens that our primer set was able to amplify quickly demonstrated that there is substantial cross-alignment among these sequences, particularly considering the large proportion of the reads that match the human sequences.

A fuller understanding of the source of cross-alignment and false positives arose from the joint analysis of the sequence results from the BBP-RMAv.2 and the MinION sequencer for any one sample. Alignment to human sequences was avoided by eliminating the human target sequences from the reference file, except one human 18S rRNA sequence. Due to the high identity between human 18S rRNA reads and the protozoan 18S rRNA sequences in the reference, false-positive cross-alignment occurred. Including a human 18S rRNA sequence in the reference file provided a higher identity target for human reads and significantly reduced the cross-alignment of human reads to pathogen targets. To further improve the specificity, the target sequences included in the reference file were reduced to the exact length of the tiles on the BBP-RMAv.2 microarray. Finally, restricting the search parameters in Minimap2 to MAPQ≥20 achieved high specificity with acceptable sensitivity (Table 5). Each NGS dataset was derived from a library prepared with the multiplex PCR products indicated by the hashtag (#) number shown in Table 5 and Tables 2, 3, indicating the microarray results for the sample with the same hashtag number. This proof-of-concept study was not extensive enough to establish a cutoff value for the alignment score that indicates a positive detection. The datasets for which Minimap2 identified the same pathogen as the microarray with alignment scores greater than or equal to 5 are likely to represent true positives. The low score and ambiguous score for dataset **#**13 make it uncertain whether *T. brucei* or *Leishmania braziliensis* has been detected. For dataset **#**14, there is no indication of a pathogen other than *S. epidermidis*; however, the low score does not permit a certainty of detection. Thus, these two results are interpreted as “possible positive.” Dataset **#**15 clearly failed to detect *Y. pseudotuberculosis.* The clean results with pathogen-negative plasma and whole blood (datasets **#**5, **#**6, and **#**16) suggest that the analysis algorithm has achieved the desired specificity.

**TABLE 5:** Pathogen identification with MinION nanopore sequencing of nucleic acid that was multiplex-amplified from pathogen-spiked human blood or plasma samples.

NGS dataset	Spiked pathogen	Conc. copies/mL	Minimap2 (MAPQ≥20)	Score	Interpretation
**#1** ^****^	CHIKV	1,000	CHIK_NSP4^††††^	269	TP^‡‡‡‡^
**#2**	Dengue virus	100	ESCO_AROE	1	FN
**#3**	HIV-1 M	10,000	HIV1 MB_GAG	23	TP
**#4**	Sindbis virus	1,000	SINDBIS_5END	16	TP
**#5**	Negative plasma		none	0	TN
**#6**	Negative plasma		none	0	TN
**#7**	*B. microti*	10,000	CCT7	11	TP
**#8**	*B. microti*	1,000	CHIK_NSP4	1	FN
**#9**	*L. aethiopica*	10,000	LEAE_ITS1-1	654	TP
**#10**	*L. aethiopica*	1,000	LEAE_ITS1-1	104	TP
**#11**	*P. vivax*	10,000	PLVI_18S_RRNA-2	8	TP
**#12**	*P. vivax*	1,000	PLVI_18S_RRNA-2	5	TP
**#13**	*T. brucei*	10,000	LEBR_ITS1-1 TRBR_18SRRNA	2 1	PP
**#14**	*S. epidermidis*	100,000	STEP_RPOB	1	PP
**#15**	*Y. pseudotuberculosis*	10,000	none	0	FN
**#16**	Negative whole blood	0	none	0	TN

^****^ Hashtag number indicates the source, listed in Tables 2, 3, of the multiplex PCR products sequenced.

^††††^ Tile sequences were compiled in a reference database. Reads were aligned with Minimap2. The tile sequences present in the reference database are shown in Supplementary Table S1, which are indicated with an asterisk (*).

^‡‡‡‡^ TP = true positive, FN = false negative, TN = true negative, FP = false positive, and PP = possible positive.

To facilitate the comparison of the BBP-RMAv.2 results to the MinION results, the outcomes for each sample are arranged side by side in Table 6. The two false negatives (datasets **#**2 and **#**15) with the MinION that were positive detections with the BBP-RMAv.2 suggest that the microarray platform is more sensitive. The *Babesia* 1,000 cells/mL result with the microarray is FN, though there were base calls on the 18SrRNA tile and FN on the MinION. We have shown from the MinION results that there is an abundant amount of human 18S rRNA in the sample that is nearly identical to *Babesia* 18S rRNA. The human fragments could hybridize to the *Babesia* 18S rRNA tile on the microarray, generating a false positive. Without confirming base calls on the *Babesia* CCT7 tile, the **#**8 interpretation is FN. The two “possible positives” in the MinION interpretation, where the result was too weak for certainty, that were both positive detections with the BBP-RMAv.2, further indicate that the BBP-RMAv.2 is more sensitive. Two false-positive results with the microarray that were interpreted as true negatives by the MinION platform suggest that NGS with the analysis algorithm that we followed is more specific than the BBP-RMAv.2. The raw MinION NGS passed reads are available at ArrayExpress at the link: (https://www.ebi.ac.uk/biostudies/) under accession number E-MTAB-14011.

**TABLE 6:** Comparison of microarray and NGS results.

Data set	Test sample	Microarray name, results (C3 score)	Interpretation	MinION Minimap2 (>20) result (score)	Interpretation
**#1**	Chikungunya virus 1,000 copies/mL plasma	CHP192, CHIKV (93)	TP	CHIKV (269)	TP
**#2**	Dengue virus type 2 100 copies/mL plasma	CHP195, DENV2 (53)	TP	*E. coli* (1)	FN
**#3**	HIV-1 Grp M 10,000 copies/mL plasma	CHP185, HIV1M (86)	TP	HIV (23)	TP
**#4**	Sindbis virus 1,000 copies/mL plasma	CHP193_re, Sindbis (100)	TP	Sindbis V. (16)	TP
**#5**	Negative control plasma	CHP207, Ld (21)	FP	None (0)	TN
**#6**	Negative control plasma	CHP211, HCV (30.5)	FP	None (0)	TN
**#7**	*Babesia microti* 10,000 cells/mL blood	CHP190, Bm (92)	TP	*B. microti* (11)	TP
**#8**	*Babesia microti* 1,000 cells/mL blood	CHP189, (0)	FN	None (0)	FN
**#9**	*Leishmania aethiopica* 10,000 cells/mL blood	CHP187, *L. aeth* (82)	TP	*L. aeth.* (654)	TP
**#10**	*Leishmania aethiopica* 1,000 cells/mL blood	CHP188, *L. aeth*. (86)	TP	*L. aeth.* (104)	TP
**#11**	*Plasmodium vivax* 10,000 cells/mL blood	CHP185_re, PLVI (88)	TP	*P. vivax* (8)	TP
**#12**	*Plasmodium vivax* 1,000 cells/mL blood	CHP186, PLVI (75)	TP	*P. vivax* (5)	TP
**#13**	*Trypanosoma brucei* 10,000 cell/mL blood	CHP183, *T brucei* (29)	TP	*T. brucei* (3)	PP^§§§§^
**#14**	*Staphylococcus epidermidis* 100K/mL blood	CHP200, *S. epi* (75)	TP	*S. epi* (1)	PP^16^
**#15**	*Y. pseudotuberculosis* 10,000 cells/mL blood	CHP198, *Y. pseudo* (43)	TP	None (0)	FN
**#16**	Negative control whole blood	CHP206, none	TN	None (0)	TN
	Scores	TP	12	TP	8
		PP	0	PP	2
		FN	1	FN	3
		FP	2	FP	0
		TN	1	TN	3
		Total	16		16

Possible Positive

## Discussion

High-density DNA resequencing microarray technology has great potential in blood safety and diagnostics. It has been shown to be effective for high-throughput detection of microorganisms in clinical, animal, and environmental samples (Leski et al., 2011; Leski et al., 2012; Berthet et al., 2013; Hans et al., 2015; Brockmann et al., 2023). The RMA platform has been validated for detection of influenza and other respiratory viruses using a large number of specimens with high sensitivity and specificity (Wang et al., 2008; Metzgar et al., 2010) and genetic disease screening (Tanner et al., 2014).

Many existing microarray-based detection assays have been developed for highly parallel pathogen screening. Most published microarrays depend on hybridization to a probe yielding a result suggesting presence/absence (Wang et al., 2002). In contrast, the BBP-RMA uses specific primers to accomplish discriminating amplification without sacrificing the sensitivity of detection, and the result of hybridization is the determination of the nucleotide sequence of the pathogen across the targeted region. The RMA represents substantially more information about the pathogen detected and achieves greater discrimination (Kourout et al., 2016).

In this study, we evaluated the BBP-RMAv.2 prototype device for detection and identification of viral, bacterial, and protozoan parasite pathogens known to pose a risk for transmission in blood and blood component transfusion. Furthermore, we developed a computer-based analysis of the microarray image to interpret the sequence revealed by the overlapping probes, score the quality of the sequences, and search high scoring sequences for matching pathogens in the sequence databases. The BBP-RMAv.2 performance was evaluated with whole human blood and plasma spiked with the infectious agents at various concentrations, challenging the system to perform with the clinical matrix.

Currently, blood safety has been improved with FDA-licensed multiplex nucleic acid tests (NATs) to screen donor plasma specimens (Dodd et al., 2020). Compared to commercial assays, the BBP-RMAv.2 has superior multiplicity to detect several gene targets from many target pathogens in one single specimen and one procedure. In addition, the results demonstrate that the BBP-RMAv.2 can detect some spiked viral pathogens at concentrations as low as 100 copies/mL, which is comparable to the limit of detection of the licensed commercial multiplex tests (which are also labeled for IU/mL with the equivalence of 1 copy per 0.56 IU (Holmes et al., 2001)) and at the viral load found in blood donor specimens. In addition, the bacterial and protozoan pathogens, for which NAT assays were recently licensed or are in development, were all detected at concentrations of 10,000 cells/mL and some as low as 100 cells/mL. Currently licensed tests for blood-borne protozoan parasites include *B. microti* (Stanley et al., 2021) and *Trypanosoma cruzi*, with a malaria test recently being licensed (FDA/CBER, 2024). However, these are all individual tests that must be applied to blood units one at a time. In contrast, BBP-RMAv.2 has been demonstrated to screen for four parasites in every sample. Furthermore, none of the available commercial assays provide pathogen-specific gene sequence information that could be used for more detailed discrimination of detected pathogen strains. The BBP-RMAv.2 would be an improvement in blood safety platforms because its high multiplicity allows low prevalence or emerging infectious agents to be included in a test panel. For example, *Plasmodium* and *Babesia*, agents whose known presence in donor blood is restricted to certain at-risk donors, could be easily included in the panel of pathogens tested more broadly with the BBP-RMAv.2.

The BBP-RMAv.2 continues the high level of multiplicity of BBP-RMAv.1 with multiple targets per pathogen genome, assuring that a mutation in a primer-binding site does not cause a failure in detection. The major improvement of BBP-RMAv.2 is the expanded list of blood-borne pathogens and tiles to achieve more discrimination of species and genotypes that can cause very different disease consequences. Version 2 also solves specific problems that were identified in the testing of version 1 (Kourout et al., 2016). For example, the limited effectiveness of the HIV-1 tile on version 1 was corrected on version 2 by 33 tiles, which can detect four major groups and eleven genotypes of HIV-1 group M.

The results of testing the BBP-RMAv.2 with 12 different pathogens led to discovery of the components of the platform needing improvement. Among viruses, HIV-1 was readily identified, but the sensitivity will need to be improved. The LTR tiles, intended to increase sensitivity, were too short, which caused a lack of specificity, suggesting that longer tiles should be designed. The Chikungunya virus was detected at the low concentration of 100 copies/mL. However, this was only possible after a manual analysis, which suggests that improvements need to be made in the bioinformatic pipeline, allowing more flexibility in the search parameters. False positives resulted from base calls just above the C3 score cutoff on two pathogen-negative plasma samples. The LESH_18SRRNA sequence result reported in the pathogen-negative plasma sample showed 24.6% identity with the tile sequence. The likely explanation is amplicon contamination, even though steps have been taken, including UTP incorporation into PCR products and strict isolation of post-PCR manipulation from the location of PCR preparation. The bases called on another negative plasma sample that matched a small segment of HCV conserved in many genotypes. The sequence result is identical to that of a primer, HCV 1A 5utr_f, with six additional 3’ bases that match the HCV genome. The interpretation of this result is not definitive, and either the HCV 1A 5utr_f primer mis-primed on the abundant human DNA in the nucleic acid sample, resulting in addition of this small fragment to the hybridization mix, or there was a very minor contamination with an HCV PCR product. These two false-positive results can be distinguished from true positives because if *Leishmania* cells or HCV particles were present, more than one of the tiles present for these species would have had base calls.

An additional component that needs improvement is the blast report for identification of the pathogen. In some cases, with low pathogen load, the sequence revealed by the microarray leads to a blast alignment that is identical in multiple organisms. The blast.report only lists the top three hits to the database. If there are more than three hits in the database that are all identical, there are no criteria for the order in which they are displayed. The best example of this unwanted result is the blast.report for *Y. pseudotuberculosis* that listed *Y. pestis* as the top hit. The two bacterial species had identical matches to the microarray base calls, but the database search arbitrarily listed *Y. pestis* first. As a minimum correction, the user must be aware to check the blast.html file, which displays all the database hits for the top scoring tile, as well as the blast.report, which only displays the top three hits. Another solution to this problem would be to re-design the *Y. pseudotuberculosis* tile to a segment that discriminates between the two bacterial species.

The microarray was manufactured with 177 independent pathogen gene sequences of 80 organisms. The 12 pathogens tested in this study covered the diverse types of pathogens, but the full capability of the BBP-RMAv.2 has not been demonstrated. The primer development process will need to continue for the BBP-RMAv.2 to reach its full final capability.

Though the BBP-RMAv.2 is a research technology, other studies have suggested that once optimized for turnaround time and cost, it can be implemented in clinical use (Davignon et al., 2005; Lin et al., 2006; Berthet et al., 2013; Hoff et al., 2021). Recognizing the limitations to throughput for the BBP-RMAv.2 suggests that using it as a supplemental test for many different screening tests may be its initial role. Modifications to the platform can increase throughput and reduce cost (Shimada et al., 2021), such that the primers and tiles that were demonstrated effective here could be more practically applied.

Understanding the current value of the BBP-RMAv.2 is also revealed by a comparison to the potential for NGS to perform similar clinical functions. To address this question directly, we performed targeted NGS with the same PCR products that were prepared for hybridization to the microarray and compared the outcomes. The Oxford Nanopore Technologies (ONT) MinION is an attractive platform due to its compact size and low initial price compared to other NGS platforms.

Beginning at the time the PCR products are produced until the platform provides an answer regarding the identity of a pathogen present in the sample, both platforms reached the answer within 24 h. However, for a fully unknown sample, the automated pipeline for the BBP-RMAv.2 was faster and simpler for the operator than the analysis pipeline for the MinION. The analysis procedure for the NGS reads required high-performance computing at a remote location and a skilled bioinformatician to reach pathogen identification. Furthermore, our results suggested that additional optimization of the sequencing procedure, read filtering, the alignment tools, and the reference file will be required to achieve the level of performance already possible with the BBP-RMAv.2. Both platforms have limitations for sample throughput. The ONT MinION flow cell, a reusable, consumable component of the MinION, processes one sample at a time, as does the Affymetrix GeneChip. If the flow cell can be washed and reused three times, the cost per sample is similar to the Affymetrix microarray. However, upon reuse, the flow cell sometimes does not have the required number of active pores. The sample throughput on the MinION is improved with barcoding, a process of ligating unique tags on the molecules in each DNA sample so that the barcoded samples can be pooled for sequencing and the reads sorted by the ONT software at the time of base calling. Thus, the number of samples per run on the MinION can be higher than that on the BBP-RMAv.2 but still lower than desired for clinical use.

The BBP-RMAv.2 microarray faces challenges in complexity and flexibility. The high multiplicity would make validation of each tile or pathogen with a separate reference test particularly burdensome because a reference sample may need to be tested simultaneously with a comparator for each of the pathogens evaluated by the BBP-RMAv.2. This problem might be solved by “a clearly defined randomized approach” to comparator testing, as recommended by the FDA Center for Devices and Radiological Health (FDA/CDRH, 2014). Additionally, detection of non-infectious contaminant nucleic acid often found in clinical specimens could impact the result interpretation (Salter et al., 2014). A weakness in flexibility exists because the manufactured RMAs cannot be changed without synthesizing a new batch of microarrays. It is a problem that would be reduced by a more modular manufacturing technique than the one employed by Affymetrix. This property of the RMA emphasizes the importance of careful design before manufacture. NGS as an approach is far superior in terms of flexibility because the platform is completely agnostic to the input DNA, providing the readout of whatever sequence is in the prepared library. In our application, we restricted the input to the material present in the PCR products and restricted the reference file to the panel of pathogens of concern. These restrictions could be adjusted without the re-manufacturing that is required for the microarray platform.

In future studies, we plan to implement some of the lessons mentioned above. The microarray would be synthesized by an alternate method that increases throughput, simplifies processing, and reduces cost (Hoff et al., 2021). The tiles arrayed on the microarray would be optimized for length to achieve more sensitive detection without sacrificing specificity. Further evaluation of the variable effectiveness of PCR primers and target tiles will facilitate primer selection to improve sensitivity. The MinION NGS approach is a usable approach. Further studies will aim to streamline the analysis pipeline. Notably, there are other pipelines such as BugSeq (Fan et al., 2021) and TaxTriage (Merritt, 2022) that could be improvements. Increasing the throughput by using barcoded library preparation with up to 24 samples in a sequencing run will also improve the NGS approach. The success in detecting the 12 pathogens tested paves the way for further pathogen testing, including clinical specimens. We have established relationships with blood collection and testing organizations that archive frozen plasma specimens from pathogen-reactive blood donations that can be tested with the BBPv.2-RMA and MinION NGS.

The BBP-RMAv.2 demonstrated a high level of multiplicity and superior sensitivity compared to the MinION targeted next-generation sequencing. The sequence analysis with the custom pipeline for the BBP-RMAv.2 was simpler and sufficiently accurate, while the bioinformatic analysis of the NGS reads was more complex and contributed to the reduced sensitivity of the MinION approach.

Protecting the blood supply from known pathogens and detecting newly emerging infectious agents remain a high priority. Today, multiplex NATs and NGS have the potential to provide valuable tools for broad-range identification of microorganisms in clinical samples. The safety of blood and blood products as well as clinical diagnosis will benefit from the continued development of new technology multiplex testing devices like the BBP-RMAv.2 and nanopore sequencing.

## Data Availability

The datasets presented in this study can be found in online repositories. The names of the repository/repositories and accession number(s) can be found below: EMBL-EBI Biostudies repository (https://www.ebi.ac.uk/biostudies/), accession numbers A-MTAB-670: https://www.ebi.ac.uk/biostudies/arrayexpress/arrays/A-MTAB-670, E-MTAB-13972: https://www.ebi.ac.uk/biostudies/arrayexpress/studies/E-MTAB-13972?query=E-MTAB-13972%20, E-MTAB-14011: https://www.ebi.ac.uk/biostudies/arrayexpress/studies/E-MTAB-14011

## Abbreviations

COA, certificate of analysis; BBP-RMA, blood-borne pathogen resequencing microarray; FDA, Food and Drug Administration; CBER, Center for Biologics Evaluation and Research; NIH, National Institutes of Health; pg, picogram.
