# Supplementary material for: Multiplex detection and identification of viral, bacterial, and protozoan pathogens in human blood and plasma using an expanded high-density resequencing microarray platform
Source: Front Mol Biosci. 2024 Jun 20;11:1419213. doi: 10.3389/fmolb.2024.1419213 (PMC11222771; doi:10.3389/fmolb.2024.1419213)
Supplement: Supplementary file 1 [file Table1.DOC]

| **TILE DETECTOR**  **Table S1. Pathogen gene segments on the BBP-RMAv.2.** Sequences of the tiles marked with an asterisk were included in the Next Generation Sequencing reference database file. | **ORGANISM NAME** | **GENE NAME** | **ACCESSION_NO: START-END*** | **LENGTH (bp)** |
| --- | --- | --- | --- | --- |
| **CTRL-TIM*** | *Arabidopsis thaliana* | TIM | NM_127687:440-905 | 466 |
| **CTRL-NAC*** | *Arabidopsis thaliana* | NAC1 | NM_104479:421-886 | 466 |
| **BABE_BAMI_CCT7*** | *Babesia microti* | CCT | LC333115:1098-1521 | 424 |
| **BABE_BAMI_18SRRNA** | *Babesia microti* | 18S rRNA | AB190459: 547:970 | 224 |
| **TRYP_LESH_18SRRNA** | *Leishmania all species* | 18S rRNA | CP029526.1: 1014849:1015372 | 524 |
| **TRYP_LEDO_ITS1-1*** | *Leishmania donovani* | ITS1 | AM901450.1:20-343 | 324 |
| **TRYP_LEDO_ITS1-2** | *Leishmania donovani* | ITS1 | OM220620.1:19-342 | 324 |
| **TRYP_LEIN_ITS1*** | *Leishmania infantum* | ITS1 | LR812960:1070152:1070475 | 324 |
| **TRYP_LEIN_CPB** | *Leishmania infantum* | Cysteine proteaseB | MW305466.1:90-363 | 274 |
| **TRYP_LEME_ITS1*** | *Leishmania mexicana* | ITS1 | AB566382:318-541 | 224 |
| **TRYP_LEME_CPB** | *Leishmania mexicana* | Cysteine proteaseB | XM_003872608.1:519-792 | 274 |
| **TRYP_LETR_ITS1-1*** | *Leishmania tropica* | ITS1 | FJ948464.1:20-343 | 324 |
| **TRYP_LETR_ITS1-2** | *Leishmania tropica* | ITS1 | FJ948455.1:20-343 | 324 |
| **TRYP_LETR_CPB** | *Leishmania tropica* | Cysteine proteaseB | DQ286773.1:519-792 | 274 |
| **TRYP_LEMA_ITS1-1*** | *Leishmania major* | ITS1 | KU680845.1:24-347 | 324 |
| **TRYP_LEMA_ITS1-2** | *Leishmania major* | ITS1 | OK374713.1:3-326 | 324 |
| **TRYP_LEMA_CPB** | *Leishmania major* | Cysteine proteaseB | KY412785.1:519-792 | 274 |
| **TRYP_LEBR_ITS1-1*** | *Leishmania braziliensis* | ITS1 | MW538634.1:42-365 | 324 |
| **TRYP_LEBR_ITS1-2** | *Leishmania braziliensis* | ITS1 | FJ753377.1:20-342 | 324 |
| **TRYP_LEMB_CPB** | *Leishmania guyanensis* | Cysteine proteaseB | GQ180933.1:519-792 | 274 |
| **TRYP_LEAE_ITS1-1*** | *Leishmania aethiopica* | ITS1 | FN677344.1:108-431 | 324 |
| **TRYP_LEAE_ITS1-2** | *Leishmania aethiopica* | ITS1 | FN677354.1:108-431 | 324 |
| **TRYP_LEAE_CPB** | *Leishmania aethiopica* | Cysteine proteaseB | HM178939.1:519-792 | 274 |
| **TRYP_TRCR_SAT*** | *Trypanosoma cruzi* | Satellite | AY520041:36-182 | 147 |
| **TRYP_TRCR_COII** | *Trypanosoma cruzi* | COX-II | MW275438.1:39-262 | 224 |
| **TRYP_TRCR_SODA** | *Trypanosoma cruzi* | Superoxide dismutase A | MZ825450.1:196-469 | 274 |
| **TRYP_TRCR_CYTB** | *Trypanosoma cruzi* | Cytochrome B | MH549665.1:98-471 | 374 |
| **TRYP_TRCR_GP1** | *Trypanosoma cruzi* | Glucose-phosphate isomerase | MN413550.1:283-656 | 374 |
| **TRYP_TRCR_18SRNA** | *Trypanosoma cruzi* | 18S rRNA | AY785579.1:400-923 | 524 |
| **TRYP_TRBR_ISG75*** | *Trypanosoma brucei* | Invariant Surface Glycoprotein | FN554968:86504:86777 | 274 |
| **TRYP_TRBR_COII** | *Trypanosoma brucei* | COX-II | M14820.1:638-1011 | 374 |
| **TRYP_TRBR_18SRRNA*** | *Trypanosoma brucei* | 18S rRNA | MN446740.1:440-963 | 524 |
| **HAEM_PL1_MITOCH*** | *Plasmodium* | maxicircle | HQ712056:597-664 | 68 |
| **HAEM_PL3_MITOCH*** | *Plasmodium* | maxicircle | KM065500:33-77 | 45 |
| **HAEM_PLFA_CYTB** | *Plasmodium falciparum* | CytB | KM032230:232-455 | 224 |
| **HAEM_PLFA_COX3*** | *Plasmodium falciparum* | COX-III | LR605957:5624-5897 | 274 |
| **HAEM_PLFA_18SRRNA-1** | *Plasmodium falciparum* | 18S rRNA | LR131462.1:2807162-2807535 | 374 |
| **HAEM_PLFA_18SRRNA-2** | *Plasmodium falciparum* | 18S rRNA | LR131462.1:2807787-2808030 | 374 |
| **HAEM_PLFA_18SRRNA-3** | *Plasmodium falciparum* | 18S rRNA | LR131487.1:1052972-1053204 | 233 |
| **HAEM_PLVI_CYTB** | *Plasmodium vivax* | CytB | KF668441:5324-5547 | 224 |
| **HAEM_PLVI_COX3** | *Plasmodium vivax* | COX-III | NC_028626.1:336-608 | 274 |
| **HAEM_PLVI_18SRRNA-1** | *Plasmodium vivax* | 18S rRNA | XR_003001206.1:844-1217 | 374 |
| **HAEM_PLVI_18SRRNA-2*** | *Plasmodium vivax* | 18S rRNA | XR_003001206:1439-1662 | 224 |
| **HAEM_PLVI_60SRRNA** | *Plasmodium vivax* | 60SrRNA | LT635620:1143396-1143769 | 374 |
| **HAEM_PLMA_18SRRNA** | *Plasmodium malariae* | 18S rRNA | XR_003751948.1:1489-1726 | 238 |
| **HAEM_PLOV-A_18SRRNA*** | *Plasmodium ovale* | 18S rRNA | LT594514.1:25796-26019 | 224 |
| **HAEM_PLOV-L_18SRRNA** | *Plasmodium ovale* | 18S rRNA | LT594514.1:25798-26021 | 224 |
| **HAEM_PLKN_18SRRNA** | *Plasmodium knowlesi* | 18S rRNA | LR701163.1:946506-946729 | 224 |
| **STAP_STAU_AT1** | *Staphylococcus aureus* | ALTX | AP017922.1:983895-984318 | 424 |
| **STAPH_STAU_MECA** | *Staphylococcus aureus* | mecA | CP033085.1:2355172-2355395 | 224 |
| **STAP_STAU_KAT*** | *Staphylococcus aureus* | Catalase | AP017922.1:1296431-1296848 | 424 |
| **STAP_STAU_HSP60** | *Staphylococcus aureus* | groL | AP017922.1:2008341-2008764 | 424 |
| **STAP_STAU_ALTX** | *Staphylococcus aureus* | EAX85 | CP033085.1:2023063-2023286 | 224 |
| **STAP_STEP_GLP** | *Staphylococcus epidermidis* | AL521 | CP014132.1:1348518-1348941 | 424 |
| **STAP_STEP_RPOB*** | *Staphylococcus epidermidis* | rpoB | CP014132.1:624446-624869 | 424 |
| **STAP_STEP_HSP60** | *Staphylococcus epidermidis* | HSP60 | FJ577634:166-389 | 224 |
| **ENTB_YEEN_HSP60** | *Yersinia enterocolitica* | HSP60 | X68526.1:578-1001 | 424 |
| **ENTB_YEEN_SODA*** | *Yersinia enterocolitica* | sodA | CP030980:47518-47941 | 424 |
| **ENTB_YEPS_HSP60** | *Yersinia pseudotuberculosis* | HSP60 | CP033715.1:1098546-1098969 | 424 |
| **ENTB_YEPS_THRA*** | *Yersinia pseudotuberculosis* | thrA | CP033715.1:2434763-2435136 | 374 |
| **ENTB_YEPS_SUCA*** | *Yersinia pseudotuberculosis* | sucA | CP033715:3157332-3157755 | 424 |
| **ENTB_ESCO_AROE*** | *Escherichia coli* | aroE | CP055256.1:486216-486439 | 224 |
| **ENTB_ESCO_GDH** | *Escherichia coli* | g-6-pdh | CP055256.1:2234646-2234869 | 224 |
| **TOGA_CHIK_NSP1** | *Chikungunya virus* | NSP1 | MW248364.1:359-532 | 174 |
| **TOGA_CHIK_NSP** | *Chikungunya virus* | NSP1 | MW248364.1:198-267 | 70 |
| **TOGA_CHIK_NSP4*** | *Chikungunya virus* | NSP4 | MW248364.1:6160-6483 | 324 |
| **TOGA_CHIK_E2** | *Chikungunya virus* | E2 | MW248364.1:8988-9311 | 324 |
| **TOGA_EEEV_NSP1** | *Eastern equine encephalitis virus* | NSP1 | OQ709280.1:128-197 | 70 |
| **TOGA_EEEV_5END** | *Eastern equine encephalitis virus* | 5’ end | OQ709280.1:4-127 | 124 |
| **TOGA_EEEV_NSP4** | *Eastern equine encephalitis virus* | NSP4 | OQ709280.1:6506-6729 | 224 |
| **TOGA_VEEV_NSP1** | *Venezuelan equine encephalitis virus* | NSP1 | MF459684.1:1062-1134 | 73 |
| **TOGA_VEEV_5END** | *Venezuelan equine encephalitis virus* | 5’ end | MF459684.1:44-167 | 124 |
| **TOGA_VEEV_NSP4** | *Venezuelan equine encephalitis virus* | NSP4 | KC344525.1:6543-6766 | 224 |
| **TOGA_SINDBIS_5END*** | *Sindbis virus* | 5’ end | MF543016.1:53-176 | 124 |
| **TOGA_SINDBIS_NSP4** | *Sindbis virus* | NSP4 | MF543016.1:6619-6842 | 224 |
| **TOGA_SINDBIS_NSP4-1** | *Sindbis virus* | NSP4 | MK045253.1:6981-7092 | 112 |
| **TOGA_SINDBIS_NSP4-2** | *Sindbis virus* | NSP4 | MK440626.1:7060-7171 | 112 |
| **FLAV_DENV_3END-1*** | Dengue virus | 3’ end | MW512367.1:10626-10694 | 69 |
| **FLAV_DENV_3END-2*** | Dengue virus | 3’ end | KY474335.1:10553-10625 | 73 |
| **FLAV_DENV1_NS5** | Dengue virus Type1 | NS5 | MF405201.1:10355-10528 | 174 |
| **FLAV_DENV1_POLYP*** | Dengue virus Type1 | Polyprotein | MF405201.1:4013-4336 | 324 |
| **FLAV_DENV2_NS5*** | Dengue virus Type2 | NS5 | MW512367.1:9819-9992 | 174 |
| **FLAV_DENV2_POLYP** | Dengue virus Type2 | Polyprotein | MW512367.1:2141-2465 | 325 |
| **FLAV_DENV3_NS5** | Dengue virus Type3 | NS5 | LC379197.1:10305-10478 | 174 |
| **FLAV_DENV3_CAP** | Dengue virus Type3 | Capsid | LC379197.1:261-585 | 325 |
| **FLAV_DENV4_NS5*** | Dengue virus Type4 | NS5 | MG601754.1:10419-10568 | 150 |
| **FLAV_DENV4_CAP** | Dengue virus Type4 | Capsid | MG601754.1:367-691 | 325 |
| **PICO_HEPAV_2C** | Hepatitis A virus | RNA Helicase | LC435031.1:4396-4619 | 224 |
| **PICO_HEPAV_3C-3D** | Hepatitis A virus | GENOME segment | LC435031.1:5633-5856 | 224 |
| **RETR_HEPBV_S*** | Hepatitis B virus | S protein | LC773631.1:1777-1976 | 200 |
| **RETR_HEPBV_C** | Hepatitis B virus | Gene X | OP611181.1:1535-1628 | 94 |
| **RETR_HEPBVA_S-P** | Hepatitis B virus, genotype A | S protein | MN080549.1:73-396 | 324 |
| **RETR_HEPBVB_S-P** | Hepatitis B virus, genotype B | S protein | MK534630.1:227-550 | 324 |
| **RETR_HEPBVC_S-P** | Hepatitis B virus, genotype C | S protein | MK720629.1:227-550 | 324 |
| **RETR_HEPBVD_S-P** | Hepatitis B virus, genotype D | S protein | OP153988.1:227-550 | 324 |
| **RETR_HEPBVE_S-P** | Hepatitis B virus, genotype E | S protein | MF772354.1:227-550 | 324 |
| **RETR_HEPBVF_S-P** | Hepatitis B virus, genotype F | S protein | KP995086.1:92-415 | 324 |
| **RETR_HEPBVG_S-P** | Hepatitis B virus, genotype G | S protein | KY004112.1:227-550 | 324 |
| **RETR_HEPBVH_S-P** | Hepatitis B virus, genotype H | S protein | KX264501.1:227-550 | 324 |
| **RETR_HEPBVR_S-P** | Hepatitis B virus, genotype I | S protein | KY471005.1:227-650 | 424 |
| **FLAV_HEPCV_5UTR** | Hepatitis C virus | 5’ untranslated | LC546833.1:22-145 | 124 |
| **FLAV_HEPCV_3UTR** | Hepatitis C virus | 3’ untranslated | MG406988.1:9592-9663 | 72 |
| **FLAV_HEPCV1_5UTR** | Hepatitis C virus, genotype 1 | 5’ untranslated | MT542088.1:128-233 | 106 |
| **FLAV_HEPCV1A_5UTR*** | Hepatitis C virus, genotype 1a | 5’ untranslated | LC368347.1:185-508 | 324 |
| **FLAV_HEPCV1B_5UTR** | Hepatitis C virus, genotype 1b | 5’ untranslated | D50480.1:276-599 | 324 |
| **FLAV_HEPCV1C_5UTR** | Hepatitis C virus, genotype 1c | 5’ untranslated | GU441323.1:82-405 | 324 |
| **FLAV_HEPCV2_5UTR** | Hepatitis C virus, genotype 2 | 5’ untranslated | KF676351.1:245-412 | 168 |
| **FLAV_HEPCV2A_5UTR*** | Hepatitis C virus, genotype 2a | 5’ untranslated | KF676351.1:287-610 | 324 |
| **FLAV_HEPCV2B_5UTR** | Hepatitis C virus, genotype 2b | 5’ untranslated | AB661417.1:59-382 | 324 |
| **FLAV_HEPCV2C_5UTR** | Hepatitis C virus, genotype 2c | 5’ untranslated | L38326.1:185-508 | 324 |
| **FLAV_HEPCV3A_5UTR** | Hepatitis C virus, genotype 3a | 5’ untranslated | KY620399.1:289-612 | 324 |
| **FLAV_HEPCV3K_5UTR** | Hepatitis C virus, genotype 3k | 5’ untranslated | GU441376.1:83-406 | 324 |
| **FLAV_HEPCV4_5UTR** | Hepatitis C virus, genotype 4 | 5’ untranslated | DQ418784.1:211-533 | 324 |
| **FLAV_HEPCV5_5UTR** | Hepatitis C virus, genotype 5 | 5’ untranslated | NC_009826.1:226-549 | 324 |
| **FLAV_HEPCV6A_5UTR** | Hepatitis C virus, genotype 6a | 5’ untranslated | KJ678813.1:246-569 | 324 |
| **FLAV_HEPGV_5UTR** | Hepatitis G virus | 5’ untranslated | AB008335.1:295-516 | 222 |
| **FLAV_WNV_3UTR-1** | West Nile virus | 3’ untranslated | MN849176.1:10506-10674 | 169 |
| **FLAV_WNV_3UTR-2** | West Nile virus | 3’ untranslated | MH939154.1:10536-10704 | 169 |
| **FLAV_WNV1_3UTR*** | West Nile virus | 3’ untranslated | MH170275.1:10545-10918 | 374 |
| **FLAV_WNV1_ENV*** | West Nile virus | Envelope | MH170275.1:1289-1662 | 374 |
| **FLAV_WNV2_3UTR** | West Nile virus | 3’ untranslated | NC_001563.2:10488-10861 | 374 |
| **FLAV_WNV_NS5** | West Nile virus | Non-structural 5 | MH170274.1:9276-9499 | 224 |
| **ORTH_AH1N1P910_HA1** | Influenza virus 2009 H1N1 | HA1 | MH329146.1:256-1131 | 876 |
| **ORTH_AH1N1P910_MA** | Influenza virus 2009 H1N1 | MA | MH329060.1:624-847 | 224 |
| **ORTH_AH1N1P910_NA** | Influenza virus 2009 H1N1 | NA1 | KJ889267:367-495/739-865 | 256 |
| **ORTH_AH1N1SE89_HA1** | Influenza virus seasonal A/H1N1 | HA1 | KP457056.1:289-1062 | 774 |
| **ORTH_AH1N1SE89_MA** | Influenza virus seasonal A/H1N1 | MA | KY926407.1:655-878 | 224 |
| **ORTH_AH1N1SE89_NA1** | Influenza virus seasonal A/H1N1 | NA1 | HQ291918:366-495/738-865 | 256 |
| **ORTH_AH3N2SE_HA3** | Influenza virus seasonal A/H3N2 | HA3 | MF955368.1:310-1068 | 759 |
| **ORTH_AH3N2SE_MA** | Influenza virus seasonal A/H3N2 | MA | MT556945.1:666-889 | 224 |
| **ORTH_AH3N2SE_NA2** | Influenza virus seasonal A/H3N2 | NA2 | MK730164.1:374-506 | 256 |
| **ORTH_AH5N1A_HA5** | Influenza virus avian A/H5N1 | HA5 | KR732525.1:314-1099 | 786 |
| **ORTH_AH5N1A_MA** | Influenza virus avian A/H5N1 | MA | MN556684.1:634-857 | 224 |
| **ORTH_AH5N1A_NA1*** | Influenza virus avian A/H5N1 | NA1 | MK392472.1:669-799 | 256 |
| **ORTH_FLUB_HA** | Influenza virus Type B | HA | MF955664.1:496-719 | 224 |
| **ORTH_FLUB_MA** | Influenza virus Type B | MA | OQ034443.1:619-842 | 224 |
| **ORTH_FLUB_NA** | Influenza virus Type B | NA | JN992784.1:221-444 | 224 |
| **ORTH_FLUC_HE** | Influenza virus Type C | HE | D63468.1:271-494 | 224 |
| **ORTH_FLUC_MA** | Influenza virus Type C | MA | CY239485.1:537-760 | 224 |
| **RETR_HIV1_LTR-1** | Retrovirus HIV-1 | Long Terminal Repeat | MH705150.1:510-575 | 66 |
| **RETR_HIV1_LTR-2** | Retrovirus HIV-1 | “ | KY658694.1:581-645 | 65 |
| **RETR_HIV1_LTR-3** | Retrovirus HIV-1 | “ | ON902104.1:1347-1412 | 66 |
| **RETR_HIV1_LTR-4** | Retrovirus HIV-1 | ‘ | MH705157.1:485-549 | 65 |
| **RETR_HIV1_LTR-5** | Retrovirus HIV-1 | ‘ | KY953200.1:8934-8999 | 66 |
| **RETR_HIV1_LTR-6** | Retrovirus HIV-1 | ‘ | MN703130.1:8978-9043 | 66 |
| **RETR_HIV1_LTR-7** | Retrovirus HIV-1 | ‘ | MH705143.1:492-9632 | 66 |
| **RETR_HIV1_LTR-8** | Retrovirus HIV-1 | ‘ | OR015504.1:4582-4647 | 66 |
| **RETR_HIV1_LTR-9** | Retrovirus HIV-1 | ‘ | OP938535.1:475-540 | 66 |
| **RETR_HIV1_LTR-10** | Retrovirus HIV-1 | ‘ | MK458196.1:1288-1353 | 66 |
| **RETR_HIV1_LTR-11** | Retrovirus HIV-1 | ‘ | MN840353.1:300-365 | 66 |
| **RETR_HIV1_LTR-12** | Retrovirus HIV-1 | ‘ | MN840352.1:289-354 | 66 |
| **RETR_HIV1_LTR-13** | Retrovirus HIV-1 | ‘ | OR015326.1:3730-3795 | 66 |
| **RETR_HIV1_LTR-14** | Retrovirus HIV-1 | ‘ | OQ092465.1:476-541 | 66 |
| **RETR_HIV1_LTR-15** | Retrovirus HIV-1 | ‘ | OP700917.1:1092-1157 | 66 |
| **RETR_HIV1_LTR-16** | Retrovirus HIV-1 | ‘ | MH705154.1:480-540 | 61 |
| **RETR_HIV1_LTR-17** | Retrovirus HIV-1 | ‘ | KY953207.1:584-646 | 63 |
| **RETR_HIV1M_P24** | HIV-1_Group M ALL Subtypes | ‘ | ON501911.1:5129-615 | 97 |
| **RETR_HIV1MA_GAG** | HIV-1_Group M subtype A | GAG | GQ432393.1:539-862 | 324 |
| **RETR_HIV1MB_GAG*** | HIV-1_Group M subtype B | GAG | KJ704793.1:661-984 | 324 |
| **RETR_HIV1MC_GAG** | HIV-1_Group M subtype C | GAG | JN014194.1:530-853 | 324 |
| **RETR_HIV1MD_GAG** | HIV-1_Group M subtype D | GAG | GQ333185.1:86-409 | 324 |
| **RETR_HIV1MG_GAG** | HIV-1_Group M subtype G | GAG | AF260492.1:82-405 | 324 |
| **RETR_HIV1MF_GAG** | HIV-1_Group M subtype F | GAG | MH705144.1:1330-1653 | 324 |
| **RETR_HIV1MH_GAG** | HIV-1_Group M subtype H | GAG | L11793.1:539-862 | 324 |
| **RETR_HIV1MJ_GAG** | HIV-1_Group M subtype J | GAG | AF082395.1:652-975 | 324 |
| **RETR_HIV1MK_GAG** | HIV-1_Group M subtype K | GAG | EF029067.1:1216-1539 | 324 |
| **RETR_HIV1CRFO1_GAG** | HIV-1_Group M subtype CRFO1 | GAG | MH327752.1:1240-1563 | 324 |
| **RETR_HIV1CRF02_GAG** | HIV-1_Group M subtype CRFO2 | GAG | AJ286370.1:139-462 | 324 |
| **RETR_HIV1N_GAG** | HIV-1_Group N | GAG | AJ006022.1:892-1215 | 324 |
| **RETR_HIV1O_GAG** | HIV-1_Group O | GAG | AF383290.1:145-468 | 324 |
| **RETR_HIV1O_P24*** | HIV-1_Group O | P24 | AY623602.1:1579-1694 | 116 |
| **RETR_HIV1P_GAG** | HIV-1_Group P | GAG | HQ179987.1:819-1142 | 324 |
| **RETR_HIV2_LTR-1** | HIV-2 all Subtypes | Long Terminal Repeat | MN942020.1:717-828 | 112 |
| **RETR_HIV2_LTR-2** | HIV-2 all Subtypes | ‘ | AB731741.1:725-836 | 112 |
| **RETR_HIV2_LTR-3** | HIV-2 all Subtypes | ‘ | MN942027.1:718-829 | 112 |
| **RETR_HIV2_LTR-4** | HIV-2 all Subtypes | ‘ | MH681611.1:9170-9259 | 90 |
| **RETR_HIV2A_P17** | HIV-2 Group A | P17 | J03654.1:547-870 | 324 |
| **RETR_HIV2B_P17** | HIV-2 Group B | P17 | EU028345.1:218-541 | 324 |
| **RETR_SIV_LTR** | Simian virus | Long Terminal Repeat | KX089791.1:135-246 | 112 |

**Table S2. Primer pairs presented in their final (2023) multiplex pools.**

| **Pool** | **Primer name** | **Sequence** | **Organism** | **Gene**  **Target** | **Amplicon Size** |
| --- | --- | --- | --- | --- | --- |
| - | Primer-Linker | CGATACGACGGGCGATCTAGC | Extension added to the 5’ end of every primer | | 21 |
| 1 | ATTIM-61F | ATGGCAGCTACCTCTCTCACTG | *Arabidopsis thaliana* | tim | **557** |
| 1 | ATTIM-NR2 | CAAGCTATCACTCCAAGACC |  |  |  |
| 1 | DENV_NS5_F4 | GAGAGACCAGAGATCCTGCTGTCT | Dengue Virus All serotypes | NS5 | 68 |
| 1 | DENV_NS5_R4 | ACCATTCCATTTTCTGGCGTT |  |  |  |
| 1 | HCV 1A 5utr_f | GCAAGACTGCTAGCCGAGTA | Hepatitis C Virus | 5’UTR | 714 |
| 1 | HCV 1A 5utr_r | TGGTGACATGGTAAAGCCCC |  |  |  |
| 1 | HEPBV_S_F4 | RTCCAGAAGAACCAAYAAGAAGATG | Hepatitis B Virus | S antigen | 199 |
| 1 | HEPBV_S_R4 | ACTCGTGGTGGACTTCTCTCA |  |  |  |
| 1 | PL_MITH_F4 | CTTACAGACGCTTCCAGAATATAACTTCT | *Plasmodium species* | mitochondrial | 97 |
| 1 | PL_MITH_R4 | GGCTGAGTCTCTATGCCTTGAAT |  |  |  |
| 1 | PLFA_COX3_F1 | CGAGTCGATCAGGAAGGTTTCATCC | *Plasmodium falciparum* | COX-III | 762 |
| 1 | PLFA_COX3_R1 | CTTGAGGCAGTTTGTTCCCTATCTACC |  |  |  |
| 1 | PLOV_18SRRNA-A_F1 | TCGTGAATATGATTTGTCTGGTTAATTCC | *Plasmodium ovale* | 18S rRNA | 311 |
| 1 | PLOV_18SRRNA-A_R1 | CGTGCAGCCTAGTTCATCTAAGG |  |  |  |
| 1 | SINDBIS_5END_F | GACCAATTGCATACCATCA | Sindbis Virus | 5” end | 253 |
| 1 | SINDBIS_5END_R | CTCGGAAAACATTCTACGAG |  |  |  |
| 1 | TRCR_SAT_F1 | ASTCGGCTGATCGTTTTCGA | *Trypanosoma cruzi* | Satellite repeat | 356 |
| 1 | TRCR_SAT_R1 | AATTCCTCCAAGCAGCGGATA |  |  |  |
| 1 | LEBR_ITS1-1_F1 | AGCAAAAGTCGTAACAAGGTAGCTGTAGG | *Leishmania braziliensis* | Internal Transcribed Spacer 1 | 409 |
| 1 | LEBR_ITS1-1_R1 | GGGGATGACACAATAGAGCTTCTCC |  |  |  |
| 1 | LEIN_ITS1_F1 | CTGGATCATTTTCCGATGATTACA | *Leishmania infantum* | Internal Transcribed Spacer 1 | 324 |
| 1 | LEIN_ITS1_R1 | TGAGAATATGGCATGCACGGGGAT |  |  |  |
| 1 | YEEN_SODA_F2 | TCCATCACACCAAACACCACCAAA | *Yersinia enterocolitica* | Super Oxide Dismutase | 495 |
| 1 | YEEN_SODA_R2 | AGAATGCTTTGATGTAGTCTGGG |  |  |  |
| 2 | ATTIM-NF1 | TGAGGGTCTTGGAGTGATAG | *Arabidopsis thaliana* | tim | 496 |
| 2 | ATTIM-1085R | GGTCGCTAACCTCCCTTTTATT |  |  |  |
| 2 | HCV 2a 5UTR_F | TGAGTGTCGTACAGCCTCCA | Hepatitis C Virus | 5’UTR | 634 |
| 2 | HCV 2a 5UTR_R | AAGCCGCACGTTAGGGTATC |  |  |  |
| 2 | LEMA_ITS1-1_F1 | CGCCCGCTTTTACCAACTTACG | *Leishmania major* | Internal Transcribed Spacer 1 | 549 |
| 2 | LEMA_ITS1-1_R1 | CGACACTGAGAATATGGCATGCACGG |  |  |  |
| 2 | LEME_ITS1_F1 | TGGTGCAATACAGGTGATCGGACAGG | *Leishmania mexicana* | Internal Transcribed Spacer 1 | 503 |
| 2 | LEME_ITS1_R1 | TGACACAATAGAGCTTCTCCC |  |  |  |
| 2 | LETR_ITS1-1_F1 | AGCAAAAGTCGTAACAAGGTAGCTGTAGG | *Leishmania tropica* | Internal Transcribed Spacer 1 | 460 |
| 2 | LETR_ITS1-1_R1 | CGACACTGAGAATATGGCATGCACGGBBVR |  |  |  |
| 2 | STEP_RPOB_F2 | TTAGCAGAGTTAACGCACAAAC | *Staphylococcus epidermidis* | RNA Polymerase B | 495 |
| 2 | STEP_RPOB_R2 | TGCTCCCATTAACGCACGGT |  |  |  |
| 2 | STAU_KAT_F1 | TCCAGAAGCATTGCACCAAGTAACG | *Staphylococcus aureus* | Catalase | 513 |
| 2 | STAU_KAT_R1 | TCGCCATATGAGAATAAACGCCCTTGC |  |  |  |
| 2 | YEPS_SUCA_F2 | GGAAACCGACATTCAGTGATGCAGA | *Yersinia pseudotuberculosis* | sucA | 559 |
| 2 | YEPS_SUCA_R2 | CCGCGAGCCTGTGACATGTTCA |  |  |  |
| 3 | ATNAC1-1158R | TAGCTGTACAATCAGACAAGCACACG | *Arabidopsis thaliana* | NAC1 | 590 |
| 3 | ATNAC1-NF1 | GGAAGACTGGGTCTTGTGTA |  |  |  |
| 3 | AH5N1A_NA_F | TTCTTACTGAGAAAGCTGTGGC | Influenza A | neuraminidase | 680 |
| 3 | AH5N1A_NA_R | ATTTGAGCCATGCCAATTATCC |  |  |  |
| 3 | BAMI_CCT7_F7 | GTGGAGCTAGAGCTAAAGGC | *Babesia microti* | CCT7 | 507 |
| 3 | BAMI_CCT7_R7 | TCCCCCTCCCACAACAATAG |  |  |  |
| 3 | CHIK_NSP4_F1 | GGAGAGTTGCCTGGACCGAGCG | Chikungunya Virus | NSP4 | 402 |
| 3 | CHIK_NSP4_R1 | ﻿TCTACTGTGAACCTATCCATTGG |  |  |  |
| 3 | DENV4_NS5_F5 | TAGCTCCGCCAATAATGGGA | Dengue Virus  Type 4 | NS5 | 178 |
| 3 | DENV4_NS5_R5 | ACCTCTAGTCCTTCCACCAG |  |  |  |
| 3 | HIV1MB_GAG_F | AGCCCAGAAGTAATACCCATGTT | HIV-1 Group M, Subtype B | GAG | 475 |
| 3 | HIV1MB_GAG_R1 | CCAACAAGGTTTCTGTCATCCAATTTT |  |  |  |
| 3 | LE_18SRRNA-1_F2 | GCCCAGCGAATGAATGACAGTAAAA | *Leishmania species* | 18S rRNA | 866 |
| 3 | LE_18SRRNA-1_R2 | AGGCCGTGAGTTGAAAAGGCGTTAC |  |  |  |
| 3 | TRBR_18SRRNA-1_R1 | CCCCGAACTACCCTCCTTCATTCC | *Trypanosoma brucei & Leishmania* | 18S rRNA | 894 |
| 3 | LE_18SRRNA-2_F2 | TCTTAGACCGCACCAAGACGAACTA | *Leishmania species* | 18S rRNA | 1158 |
| 3 | LEDO_ITS1_R4 | TTACTGCAAATTTTGAGTACAAAAC |  |  |  |
| 3 | LEDO_ITS1_F4 | AAACATATACAACTCGGGGAGA | *Leishmania donovani* | Internal Transcribed Spacer 1 | 91 |
| 3 | DENV1_POLYP_F1 | AATCTAGTCAAATCAATGGTCTC | Dengue Virus  Type 1 | Polyprotein | 962 |
| 3 | DENV1_POLYP_R1 | GTGTCATCTCTCTCTTCATC |  |  |  |
| 4 | ATNAC1-84F | AAATCATGGAGACGGAAGAAGA | *Arabidopsis thaliana* | NAC1 | 558 |
| 4 | ATNAC1-NR2 | AGCTTCCCATGTTGTCTCTA |  |  |  |
| 4 | DENV2_NS5_F11 | ﻿TTGGTAGGGCCCGAATTTCCCA | Dengue Virus  Type 2 | NS5 | 365 |
| 4 | DENV2_NS5_R7 | CCTGCTTGTTAGCCCAATCA |  |  |  |
| 4 | ESCO_AROE_F1 | ACCCTCAAGCGGTTAGAAGATGG | *Escherichia coli* | aroE | 332 |
| 4 | ESCO_AROE_R1 | GGAATATCACCACTGATGCCACTGG |  |  |  |
| 4 | HIV_p24_F | GGACACCAAGGGGCTTTACA | HIV-1 Group O | P24 | 454 |
| 4 | HIV_p24_R | AGGTAGCTCCTGGCCCTAAT |  |  |  |
| 4 | LEDO_ITS1-1_F1 | CTGGATCATTTTCCGATGATTACA | *Leishmania aethiopica* | Internal Transcribed Spacer 1 |  |
| 4 | LEDO_ITS1-1_R2 | CTTCTCCCATGCGCCGTTTGC |  |  |  |
| 4 | TRBR_ISG75_F2 | CCTCAGCCTCCTCCAAAGCCTTTCT | *Trypanosoma brucei* | TbgDal_V310 | 987 |
| 4 | TRBR_ISG75_R1 | CAACGATGCCTGTTACATTACG |  |  |  |
| 4 | WNV1_ENV_F2 | CAACGGCTGCGGACTATTTG | West Nile Virus | env | 869 |
| 4 | WNV1_ENV_R2 | GTTGTTCTCCTCTGCCCACCACTAT |  |  |  |
| 4 | YEPS_THRA_F1 | CGTATCGGTGCCAGCGGTATTCC | *Yersinia pseudotuberculosis* | thrL | 1487 |
| 4 | YEPS_THRA_R1 | GGTTCGGTATACCCCAACGCCTTAGC |  |  |  |
